# Spatial variations in health service utilization among migrant population: a perspective on health equity

**DOI:** 10.3389/fpubh.2024.1447723

**Published:** 2024-10-08

**Authors:** Dan Li, Masaaki Yamada, Dawei Gao, Feifan Yang, Haisong Nie

**Affiliations:** ^1^United Graduate School of Agricultural Science, Tokyo University of Agriculture and Technology, Tokyo, Japan; ^2^Division of International Environmental and Agricultural Science, Institute of Agriculture, Tokyo University of Agriculture and Technology, Tokyo, Japan; ^3^College of Economics and Management, Zhengzhou University of Light Industry, Zhengzhou, Henan, China; ^4^Joint Doctoral Program for Sustainability Research Graduate School of Engineering, Tokyo University of Agriculture and Technology, Tokyo, Japan

**Keywords:** migrant population, health service utilization, health equity, spatial variation, geodetector, China

## Abstract

As health equity becomes a prioritized goal in global health policy, extensive research has revealed that socio-economic and geographical factors jointly exacerbate barriers to medical service access for both internal and international migrant populations, further accelerating existing health disparities. This study explores healthcare service utilization disparities among internal migrants in China, a population profoundly affected by the country’s economic reforms and urbanization since the late 1970s. These transformations have led to significant migratory movements and subsequent healthcare challenges for these populations. Leveraging data from the 2017 China Migrant Dynamic Survey, comprising 169,989 samples across 28 provinces, we introduce a novel metric—the “No Treatment ratio” (NT-ratio). This ratio quantifies the proportion of migrants who, after falling ill, choose not to seek treatment relative to the total migrant population in a given province or region, serving as a critical measure of health risk. Building upon Anderson’s Behavioral Model of Health Services Use, we adapted the model to better reflect the unique circumstances of migrant populations. The study employs spatial autocorrelation, hotspot analysis, and geodetector techniques to dissect the multifaceted factors influencing healthcare disparities. Our Findings reveal that the NT-ratio is significantly higher in eastern and northeastern China. Key factors influencing the NT-ratio include age, left-behind experiences, health education, and *per capita* medical resources. In response to these disparities, we recommend optimizing the distribution of medical resource, strengthening tiered diagnosis and treatment systems, and integrating health, education, and social security resources. These measures aim to improve healthcare utilization among migrant populations and reduce health inequities, aligning with global health objectives.

## 1 Introduction

Health equity is a vital benchmark for gauging the efficacy of a nation’s health service policies and reform initiatives. Prioritizing equitable health service access and systems to safeguard vulnerable populations is now critical in global policy agendas. Cultural, linguistic, racial, and socioeconomic factors fuel persistent health inequities. Extensive research has illuminated the underlying mechanisms of these inequalities within countries (1–6). Disparities in medical care access, particularly for disadvantaged groups including migrants, have garnered increased attention. In China, health inequity reflects not just differences in health outcomes, but more deeply, the unequal distribution of social resources and opportunities, a disparity that is especially marked among internal migrants.

In the late 1970s, China began implementing its “reform and opening-up” policy, transitioning from a centrally planned economy to a market-oriented economy while integrating into the global economy. Progressive economic reforms have steadily boosted urban labor demands. In response, surplus rural laborers have undertaken extensive migrations in search of better educational and employment opportunities. Data from the National Bureau of Statistics’ Seventh National Census Bulletin (No. 7) show that as of November 2020, China’s internal migrant population had reached 375,816,759, making up 26.65% of the total population (1.41 billion). This represents an increase of 154,390,107 individuals, or 69.73%, compared to 2010. Development models focused on economic outcomes frequently overlook the health concerns of the migrant population. Although the “healthy migrant hypothesis” suggests that migrants generally enjoy better health than natives, studies have also identified the “salmon bias hypothesis” within China’s internal migrant population. This bias explains why the self-assessed health of migrants moving from rural areas to cities often seems better than that of those who remain in rural areas or return to their hometowns after migrating. Migrants whose health deteriorates due to factors such as living costs and social security needs often cannot remain long-term in destination cities and choose to return to their original homes (7). Therefore, ensuring that migrants have equal access to healthcare services in their current residences is crucial for improving their health outcomes.

Following the implementation of new medical reforms, China has committed to fundamentally improving the household registration system and addressing the disparities in public services it engenders. In 2013, the government introduced the “Pilot Work Plan for the Equalization of Basic Public Health and Family Planning Services for the Migrant Population”.^1^ This initiative has effectively enhanced the health of the migrant population by improving access to medical services, health literacy, and medical service utilization (8). It has also broadened the migrant population’s understanding of policies and social insurance, further boosting their use of medical services (9). However, for the migrant population, the improvement of overall medical accessibility does not mean the equality of medical service utilization. Zhou et al. (8) found that the equalization pilot policies primarily benefited high-income groups, thus intensifying healthcare usage disparities within the migrant population.

The underutilization of basic public health services among the migrant population is linked to geographic distribution (10). Social factors (11), individual predisposition, enabling characteristics, and need-based factors also play significant roles (12–14). Furthermore, the total availability and allocation of medical resources, service quality, and the effectiveness of community health services are key determinants of the health status of the migrant population (15). Therefore, achieving health equity entails eliminating disparities in health outcomes stemming from socioeconomic, demographic, or geographic differences—a fundamental right that ensures everyone can fully realize their potential for health and well-being (16). This calls for a comprehensive and systematic examination of the factors influencing medical service utilization among the migrant population.

However, existing research on healthcare utilization among the migrant population exhibits several limitations. First, research on migrant health service utilization predominantly employs Anderson’s Behavioral Model of Health Services Use (BMHSU) (17–19), crucial for policy enhancement but limited for Chinese migrant studies. The range of influencing factors proposed by BMHSU may not comprehensively account for the unique circumstances of China’s migrants. Empirical evidence suggests that besides BMHSU’s factors, variables like migration time and distance (11, 20), individual life experiences (21), and family resource endowment significantly impact migrants’ health service utilization. Second, many studies fail to consider all relevant influencing factors. Migrants are diverse, differentiated by migration scope and duration, left-behind experiences^2^, family economic status, educational background, and more. Policies to improve health service access need to account for these sub-groups’ varied needs. Additionally, many studies view health service utilization as primarily driven by individual choices, neglecting the influence of external environmental factors like regional variations (9, 13, 22). Health outcomes are deeply influenced by broader social, economic, and environmental contexts (23). Thus, incorporating a spatial dimension in examining health service utilization among migrants is vital for more effective policy development and implementation.

Therefore, we develop a refined model tailored for analyzing medical care utilization by internal migrants. This model builds on the BMHSU and prior studies, incorporating a broader perspective and additional influencing factors. We employs a multidimensional geographic framework, including spatial autocorrelation, hotspot analysis, and the geodetectors, to assess the spatial distribution of contextual and individual factors and their interactions in medical care utilization. This study addresses a gap in previous research, which primarily focused on individual and social determinants. It aims to elucidate spatial disparities in health service access among migrants and to identify key factors contributing to underutilization and provide policymakers with actionable insights for more targeted health service interventions.

The core issue this study seeks to address is:

1. Are there spatial disparities in healthcare utilization among internal migrants?
2. What are the individual and contextual factors influencing healthcare utilization among internal migrants?
3. How can these insights inform policy decisions to reduce healthcare inequities for migrant populations?

## 2 Methods

### 2.1 Data source and variables

#### 2.1.1 Data source

The spatial statistical data for this study were derived from the 2017 China Health and Family Planning Statistical Yearbook and the 2017 China City Statistical Yearbook. The former provides comprehensive statistical data on health service development and resident health levels across China’s 31 provinces and regions, encompassing 16 key aspects such as health institutions, facilities, funding, public health status, and medical insurance coverage. The latter offers detailed provincial and regional data on air quality and pollution emissions.

Data on the migrant population was obtained from the 2017 China Migrant Dynamic Survey (CMDS), covering the entire migrant population across 31 Chinese provinces, including the Xinjiang Production and Construction Corps, for its primary sampling frame. The CMDS 2017 targeted non-local registered migrants aged 15 and above from cities and counties, using a Probability Proportional to Size (PPS) sampling technique. The survey amassed 169,989 migrant responses. With the aim of evaluating health service utilization among Chinese internal migrants, our focus was on those who reported illness or physical discomfort in the past year. After removing samples that were only “cross-county within the same city” migrants and those with incomplete data, 61,978 samples were subjected to analysis.

#### 2.1.2 Dependent variable

Question Q411 in the questionnaire, “Where did you first seek medical care the last time you were sick, injured, or felt unwell?”. This question assumes the respondents’ perception that their illness, injury, or physical discomfort warranted some form of medical care. The question is designed to capture a comprehensive range of healthcare-seeking behaviors, not limited to hospital visits, and to identify respondents’ initial choices of care, whether that involved visiting a hospital, clinic, pharmacy, or choosing not to seek any medical care at all. Table 1 presents the distribution and percentage of participants engaging in each behavior.

**Table 1:** Distribution of primary health service utilization among migrants.

No.	Health service utilization	Abbreviation	Sample	Proportion
1	Local Community public health station	CPHS	12,439	20%
2	Local Private clinic	PC	8,741	14.1%
3	Local General/Specialist Hospital	G/SH	10,470	16.8%
4	Local Pharmacy	LP	19,351	31.2%
5	Nowhere, no treatment	NT	10,971	17.7%

Among the behavioral options, choosing 1. Community Public Health Services (CPHS); 2. Primary Care (PC); or 3. General/Specialist Hospital (G/SH) represents a more advisable approach to medical care. Option 4, Pharmacy, some migrant patients opt for self-medication by purchasing drugs from pharmacies, based on their own illness experiences. However, option 5, No Treatment (NT), carries a high health risk and, if overlooked, could significantly deteriorate an individual’s health and affect the migrant population’s overall health. Therefore, we introduces the concept of the “No Treatment ratio” (NT-ratio), defined as the proportion of migrants who opt not to seek treatment after falling ill out of the total migrant population in a region/province, as the dependent variable to identify region healthcare disparities.

#### 2.1.3 Independent variable

BMHSU emphasizes contextual factors in recognition of the importance of community, the structure and process of providing care (24), and the realities of a managed care environment (25). Building on the BMHSU, this study delves into the various factors influencing health service utilization among the migrant population, including both contextual and individual characteristics. Contextual characteristics encompass macro-socioeconomic factors include Beliefs, Financing, Organization, Population Health indices, and Environmental conditions.

Individual characteristics include Demography, Social, Beliefs, Financing, Organization, and Perceived health status. Additionally, after conducting a comprehensive analysis of China’s policy landscape and the demographic characteristics of migrants, we have incorporated mobility attributes into the Predisposing dimension of the BMHSU. The factors and rationale encompassed are below:

#### 2.1.4 Migrant characteristics

##### 2.1.4.1 Type of migrant

The types of internal migrants in China are cross-province, cross-city within the province, and cross-county/district within the city. The problem of cross-regional treatment caused by mobility has become an essential obstacle for migrants seeking medical treatment, leading to the contradiction between the availability of medical resources in non-insured areas and the medical needs of migrants.

##### 2.1.4.2 Left-behind experience

**Figure 1:**
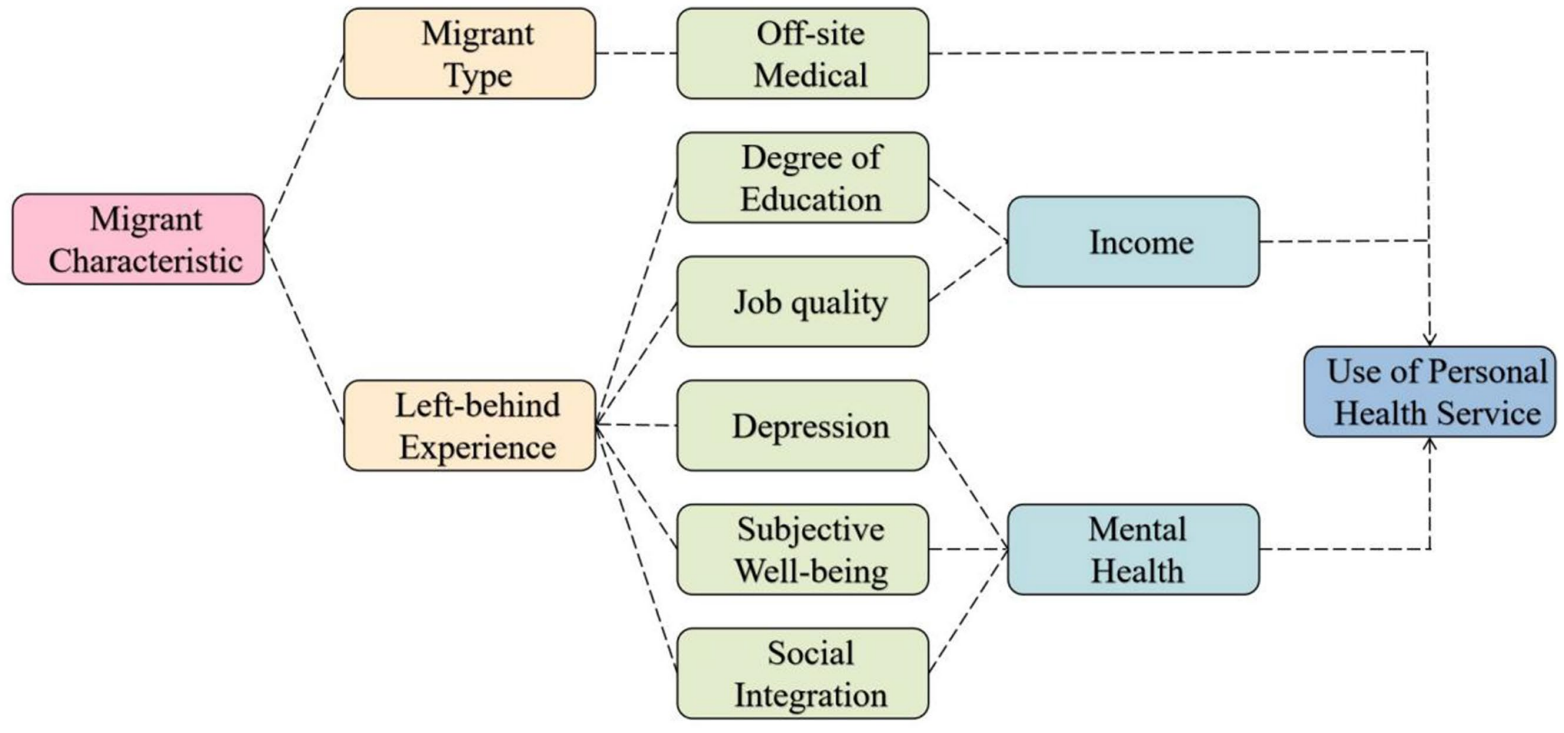
Pathways of health service use influenced by migrant characteristics.

In 2020, there were 66.93 million left-behind children in China, and a total of 138 million children were affected by population mobility, accounting for 46.4% of the total child population (26). Research indicates that the effects of left-behind experience (LBE) are comprehensive, spanning from childhood into adulthood. Key findings reveal that left-behind children exhibit significantly lower levels of positive mental health metrics—such as happiness, life satisfaction, and prosocial behavior—compared to their non-left-behind rural counterparts^3^ (27). This discrepancy extends into reduced educational opportunities upon reaching high school. In adulthood, LBE correlates with increased depression and job mobility, leading to diminished social integration and job quality (28, 29). These dynamics adversely affect the socioeconomic and mental health status of migrants, contributing to health disparities within the group and altering their health service utilization. Consequently, this study proposes a model suggesting that migrant characteristics significantly influence health service utilization patterns among migrants (Figure 1).

We utilized Self-Rated Health (SRH) and Chronic Diseases (CD) as indicators to assess both the subjective perceptions and objective realities of migrants’ health, along with associated risks. Additionally, the participation rate in health education programs (Participation in Health Education, PHE) and the coverage of Resident Health Records (RHR) were analyzed across different provinces and regions to gauge the level of understanding and awareness of health-related policies among the migrant population.

Tables 2, 3 provide detailed descriptions of the variables and abbreviations used in the modified BMHSU in this study, including specific definitions and measurement methods for both contextual and individual characteristics.

**Table 2:** Description of contextual characteristic variables.

Types		Variables	Description
Dependent variable		No treatment ratio (NT-ratio)	The ratio of migrant population not seek treatment in each province to the total diseased migrant population.
Independent variable			
Predisposing	Beliefs	Number of participants in health education (PHE)	The number of health education participants per 1,000 population by province
Enabling	Financing	*Per capita* disposable income (PCDI)	*Per capita* disposable income by province
		Total health expenditure *per capita* (PTHE)	The ratio of total health expenditure to the average population during the same period by province in 2016
		Medical insurance coverage rate for urban employees basic insurance coverage rate (UEBMI)	The participation rate of basic medical insurance for urban employees by provinces (%)
		*Per capita* disposable income for health care (PCDIHC)	*Per capita* disposable income for health service by province (CNY)
	Organization	Number of hospitals per thousand population (NH)	Number of hospitals per thousand population by province
		Number of basic medical institutions (NBMI)	Number of basic medical institutions per 1,000 population by province
		Number of general practitioners (NGP)	Number of general practitioners per 1,000 population in each province
		Number of hospital beds (NHB)	Number of hospital beds per 1,000 population by province
Need	Population Health indices	Population life expectancy (PLE)	Population life expectancy by province
		Provincial mortality rate (PMR)	Mortality rate by province
	Environmental	Provincial annual precipitation (PAP)	Annual precipitation by province
		Industrial Waste Water Discharged (IWWD)	Annual industrial wastewater discharge by province (ton)
		Household Waste Water Discharged (HWWD)	Annual domestic wastewater discharge by province (ton)

**Table 3:** Description of individual characteristic variables.

Types		Variables	Description
Independent variable			
Predisposing	Demography	SEX	Proportion of male migrant population
		AGE	Average age of migrant population in each province
	Social	Marital status (MARRY)	Proportion of migrant population without spouse
		Education (EDU)	Average years of education of the migrant population
		Social Network (SN)	The proportion of migrant population with minimal social interactions
	Beliefs	Health Education (HE)	The proportion of the migrant population that has never received any health education
		Resident Health Record (RHR)	The proportion of the migrant population without a resident file established in the community
	Migrant characteristics	Trans-provincial migrant (TPM)	The proportion of trans-provincial migrant population to the total migrant population by province
		Social integration (SI)	The proportion of migrants who subjectively feel unable to integrate into the inflow city by province
		Left Behind Experience (LBE)	The proportion of the migrant population with experience of being left behind
		Inflow time (TIME)	Average inflow time of the migrant population by province.
Enabling	Financing	Income expenditure ratio (IE-ratio)	Income expenditure ratio of migrant population by province
		Monthly income (INCOME)	Average monthly income of migrant population by province
		New rural cooperative medical service (NRCMS)	The participation rate in the New Rural Cooperative Medical Scheme among the migrant population by province (%)
	Organization	Accessibility to Medical Care (AMC)	The proportion of the migrant population living less than 15 min away from the nearest medical facility
Need	Perceived	Self-rated Health (SRH)	Proportion of migrants who consider themselves healthy
		Chronic Disease (CD)	The proportion of the migrant population without chronic diseases by province

### 2.2 Research technique

#### 2.2.1 Spatial autocorrelation statistics

*Moran’s I* statistics Moran’s *I* is an index used to measure spatial autocorrelation, reflecting the degree of similarity in attribute values between neighboring spatial units. It incorporates two forms of testing: random distribution and normal distribution. Equation 1 is the formula for Moran’s *I* is:

(1) $$Moran^{'}sI=\frac{\sum_{i=1}^{n}\sum_{j=1}^{n}w_{ij}\left(x_{i}-\bar{x}\right)\left(x_{j}-\bar{x}\right)}{s^{2}\sum_{i=1}^{n}\sum_{j=1}^{n}w_{ij}}$$

In this formula: $\bar{x}=\frac{\sum_{i=1}^{n}x_{i}}{n};s^{2}=\frac{\sum_{i=1}^{n}{\left(x_{i}-\bar{x}\right)}^{2}}{n};x_{i},x_{j}$ represent the observed values of the NT-ratio for province *i* and *j*, respectively. “*n*” denotes the number of provinces, and “*wij*” is the spatial weight matrix, indicating whether provinces *i* and *j* are spatially adjacent. If Moran’s *I* is greater than 0, it indicates the presence of positive spatial autocorrelation, suggesting that the NT-ratio tends to be spatially clustered. Conversely, if Moran’s *I* is less than 0, it indicates negative spatial autocorrelation, implying that the NT-ratio tends to be spatially dispersed.

We employs LISA (Local Indicators of Spatial Association) cluster maps to display four types of local spatial associations, identified through Moran’s normal distribution significance tests. These types are categorized based on the sign of the *I_i_* value (Equation 2). Areas with *I_i_* > 0 indicate positive local spatial autocorrelation, classified as “High-High” and “Low-Low” regions. Conversely, areas with *I_i_* < 0, indicating negative local spatial autocorrelation, are classified as “Low-High” and “High-Low” regions.

(2) $$I_{i}=\frac{\left(x_{i}-\bar{x}\right)}{s^{2}}\overset{n}{\underset{j=1,j\neq 1}{\sum }}w_{ij}\left(x_{i}-\bar{x}\right)$$

#### 2.2.2 Getis-Ord Gi* statistic

The Getis-Ord Gi* statistic primarily investigates spatial patterns by calculating the relationship between a geographic attribute at a specific location and those of its neighboring locations. It identifies whether spatial elements belong to clusters of high or low values. Although the LISA scatter plot, corresponding to Moran’s *I*, also assesses the similarity or dissimilarity of spatial data, it does not distinguish the type of spatial clustering pattern. In contrast, the Getis-Ord Gi* statistic, based on a normal distribution hypothesis test, demonstrates greater sensitivity than LISA, which relies on a random distribution hypothesis test (30), which is based on a random distribution hypothesis test. This enhanced sensitivity of Getis-Ord Gi* enables the detection of whether regional units exhibit high-value or low-value clustering. Hotspot analysis (Getis-Ord Gi*) is employed to identify spatial clusters of statistically significant high values (hot spots) and low values (cold spots) in the NT-ratio. This is achieved by calculating the Getis-Ord Gi* value for each element, resulting in a z-score and *p*-value for each (31, 32). Equation 3 is the Getis-Ord Gi* local statistic formula.

(3) $$G_{i*}=\frac{\overset{n}{\underset{j=1}{\sum }}w_{i,j}-\bar{x}\overset{n}{\underset{j=1}{\sum }}w_{i,j}}{\sqrt[{s}]{\frac{n\overset{n}{\underset{j=1}{\sum }}w_{i,j}-\left(\overset{n}{\underset{j=1}{\sum }}w_{i,j}\right)}{n-1}}},\bar{x}=\frac{\overset{n}{\underset{j=1}{\sum }}x_{i}}{n},s=\sqrt{\frac{\overset{n}{\underset{j=1}{\sum }}x_{i}^{2}-{\left(\bar{x}\right)}^{2}}{n}}$$

In the formula: “*x_j_*” denotes the attribute value of province j; “*w_i，j_*” represents the spatial weight between provinces *i* and *j*; “*n*” is the total number of provinces; $\bar{x}$ is the mean of the NT-ratio; “*S*” is the standard deviation of the NT-ratio. The Gi* statistic is calculated as a z-score. A higher z-score indicates tighter clustering of high values (hot spots), whereas a lower z-score indicates tighter clustering of low values (cold spots) (33).

#### 2.2.3 Geodetector

Geodetector is a suite of statistical methods designed to detect spatial heterogeneity and to reveal the driving forces behind it. Its core premise is based on the hypothesis that if an independent variable significantly influences a dependent variable, then the spatial distributions of the independent and dependent variables should exhibit similarities (34, 35). The unique advantage of the geodetector lies in its ability to identify risk factors affecting the distribution of the NT-ratio, as well as to detect how two factors interact to influence the NT-ratio. This is achieved by calculating and comparing the *q*-values of individual factors and the combined *q*-value after overlaying two factors. Such an analysis allows for the determination of whether an interaction between two factors exists and the nature of this interaction in terms of its strength, direction, and whether it is linear or non-linear (36).

#### 2.2.4 Factor detection

Investigates the spatial variability of the attribute *Y* and the extent to which a specific factor *X* accounts for this spatial variability of *Y*. The quantification is achieved using the *q*-value, expressed as follows:

In the formula (Equation 4), *h* = 1, …, *L* represents the strata of variable *Y* or factor *X*，which refers to classification or partitioning; *N_h_ and N* are the number of units in stratum *h* and the entire area: $\sigma_{h}^{2}$ and $\sigma^{2}$ are the variances of *Y* values within stratum *h*and the entire area, respectively. *SSW* and *SST* (Equation 5) represent the within Sum of Squares and the total Sum of Squares. The range of *q* is [0, 1], with higher values indicating more pronounced spatial differentiation of *Y;* if the stratification is generated by the independent variable *X*, a higher *q* value suggests a stronger explanatory power of *X* over attribute *Y*, and vice versa. In extreme cases, *q* = 1 indicates that factor *X* completely controls the spatial distribution of *Y q* = 0 indicates that factor *X* has no relation to *Y*, and the *q* value represents that *X* explains 100 × *q*% of *Y*.


(4) $$q=1-\overset{L}{\underset{h=1}{\sum }}N_{h}\sigma_{h}^{2}=1-\frac{SSW}{SST}$$

(5) $$SSW=\overset{L}{\underset{h=1}{\sum }}N_{h}\sigma_{h}^{2},SST=N\sigma^{2}$$

#### 2.2.5 Interaction detection

Identifying the interactions between different risk factors *Xs*, that is, assessing whether the combined effect of factors *X_1_* and *X_2_* increases or decreases the explanatory power over the dependent variable *Y*, or whether their effects on *Y* are independent of each other. The evaluation method is to first calculate the *q* values of the two factors *X_1_* and *X*_2_ on Y separately: *q*(*X_1_*) and *q*(*X_2_*), then calculate the *q* value when they interact: *q*(*X_1_*∩*X_2_*), and compare *q*(*X_1_*), *q*(*X_2_*), and *q*(*X_1_*∩*X_2_*). The relationship between the two factors can be categorized as Table 4.

**Table 4:** Basis for judging two-factor interaction patterns.

Type	Basis of judgment	Interaction
1	*q*(*X_1_* ∩ *X_2_*) < *Min*(*q*(*X_1_*), *q*(*X_2_*))	Nonlinear weakening
2	*Min*(*q*(*X_1_*), *q*(*X_2_*)) < *q*(*X_1_*∩*X_2_*) < *Max*(*q*(*X_1_*), *q*(*X_2_*))	Single nonlinear enhancement
3	*q*(*X_1_*∩*X_2_*) < *Max*(*q*(*X_1_*), *q*(*X_2_*))	Double enhancement
4	*q*(*X_1_*∩*X_2_*) = *q*(*X_1_*) + *q*(*X_2_*)	Independence
5	*q*(*X_1_*∩*X_2_*) > *q*(*X_1_*) + *q*(*X_2_*)	Nonlinear enhancement

## 3 Results

### 3.1 Descriptive statistics

**Figure 2:**
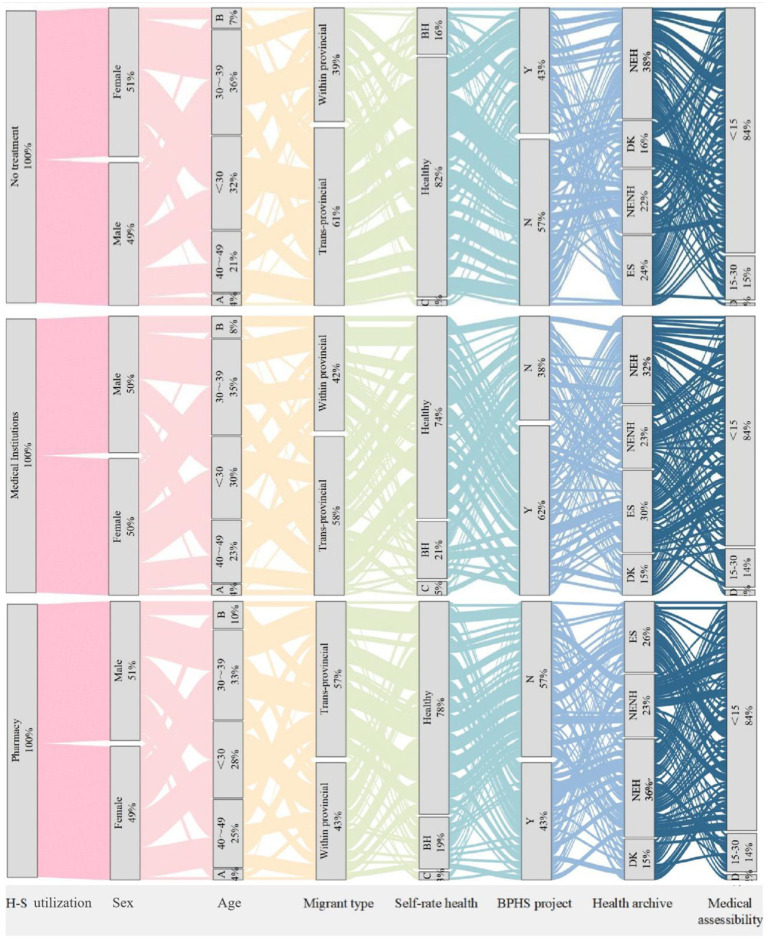
Sankey diagram of basic health service utilization among migrant population. H-S utilization, health service utilization; A: aged more than 60 years; B: 50~59; BH, basic health; C: ill health; BPHS project: Have you heard of the "National Basic Public Health Services Program"? DK: I don’t know; NEH: No, it has not been established, but I have heard of it; NENH: No, it has not been established, and I haven’t heard of it; ES: Yes, it has been established; D: more than 30 minutes.

Migrants’ illness incidence within 2 weeks is 6.47% (10,996/169,989), while the annual illness rate is 48.68% (82,744/169,989). We employed Sankey diagrams (Figure 2) to depict the health status and awareness of health policies among 61,978 ill migrants. Analysis of questionnaire data on initial medical consultation preferences showed varied choices: 19.92% selected community health centers, 14.10% chose private clinics, 16.89% went to general/specialized hospitals, 31.3% preferred pharmacies, and 17.70% avoided treatment. Pharmacies were the most common choice for post-illness or injury health service among migrants.

Further examination grouped community health stations, private clinics, and hospitals as formal medical institutions, contrasting these with pharmacy visits and no treatment. The demographic of ill migrants was evenly distributed by gender, with the majority aged between 20 and 40 years old. The migrant population opting for no treatment exhibited the highest rate of cross-provincial mobility at 60%, with the highest self-rated health status, where 82% perceived themselves as healthy. This contrasts with a slightly lower rate of 78% for those choosing local pharmacies, while migrants seeking care from medical institutions reported the lowest self-perceived health status at 74%. The findings suggest that migrants with poorer self-reported health are more likely to seek care from formal medical institutions, indicating a stronger perception of illness severity. In contrast, trans-provincial migrants may avoid formal medical services due to time constraints (37) or greater economic pressures. Similar to international migrants, internal migrants are also influenced by changes in their living environment, lifestyle, and cultural differences (38). Although they often report better self-rated health, this perception may not accurately reflect their true health status, as limited access to healthcare resources can lead to an underestimation of health risks. The proximity to medical facilities within 15 min was similar across groups, standing at 84%, indicating no significant disparities in healthcare access. Knowledge of national public health programs was most prevalent among individuals using formal medical institutions at 62%, marking a 4 and 6% greater establishment of health records than those opting for pharmacies or no treatment, respectively. This suggests that national level policy intervention can facilitate more appropriate health service utilization among migrant populations.

**Figure 3:**
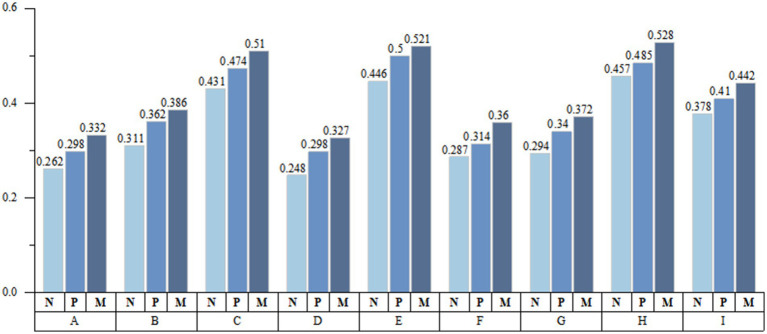
Health education participation of migrant population. A: occupational disease prevention and control; B: STD/AIDS prevention and treatment; C: reproductive health and contraception; D: tuberculosis prevention and cure; E: smoking control; F: mental health; G: chronic disease prevention; H: maternal and child health care; I: save yourself from public emergencies; N: no treatment; P: pharmacy; M: medical institution.

The Health Outcome Model emphasizes health literacy as a key outcome of health education. By improving access to health information and enhancing the ability to use it effectively, improving health literacy is essential for empowerment (39). In our comparative analysis of health education participation among ill migrants receiving institutional treatment, pharmacy treatment, and no treatment, we observed that those who chose institutional treatment were more actively engaged in all types of health education, illustrates that health education is an effective approach to guiding the migrant population towards appropriate healthcare utilization, consistent with previous international research findings (40, 41). However, this engagement is not uniformly distributed across health education areas. The highest levels of engagement were in Reproductive health and contraception (C), Smoking control (E), and Maternal and child health care (H). Conversely, their participation was notably lower in occupational disease prevention and control (A), tuberculosis prevention and cure (D), mental health (F), and chronic disease prevention (G) (Figure 3).

**Figure 4:**
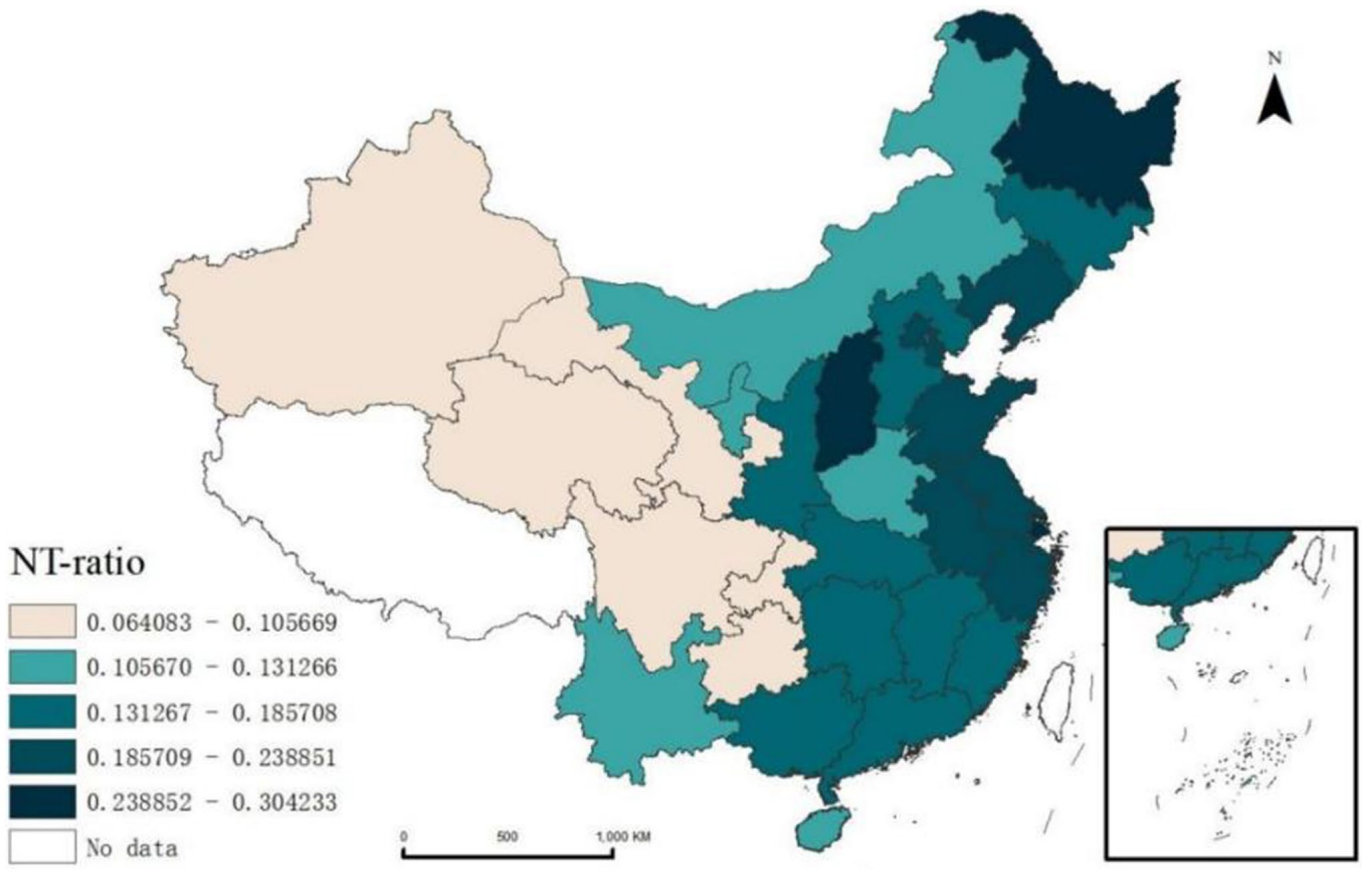
Spatial distribution of NT-ratio among migrants in China.

Spatial statistical analysis reveals significant regional variations in the NT-ratio among China’s migrant population. Shanghai has the highest NT-ratio at 30.42% (Figure 4). The Hu Line^4^ illustrates a distinct divide, with western regions showing lower NT-ratio compared to the eastern regions. This pattern indicates that regional economic and cultural factors play a crucial role in health service utilization among migrants. In developed eastern areas, despite better health service infrastructure, high living costs and fast-paced lifestyles may hinder migrants’ access to timely health service. Conversely, the less developed western regions have lower NT-ratio, due to lower population densities that allow for stronger community support networks.

### 3.2 Spatial autocorrelation

**Figure 5:**
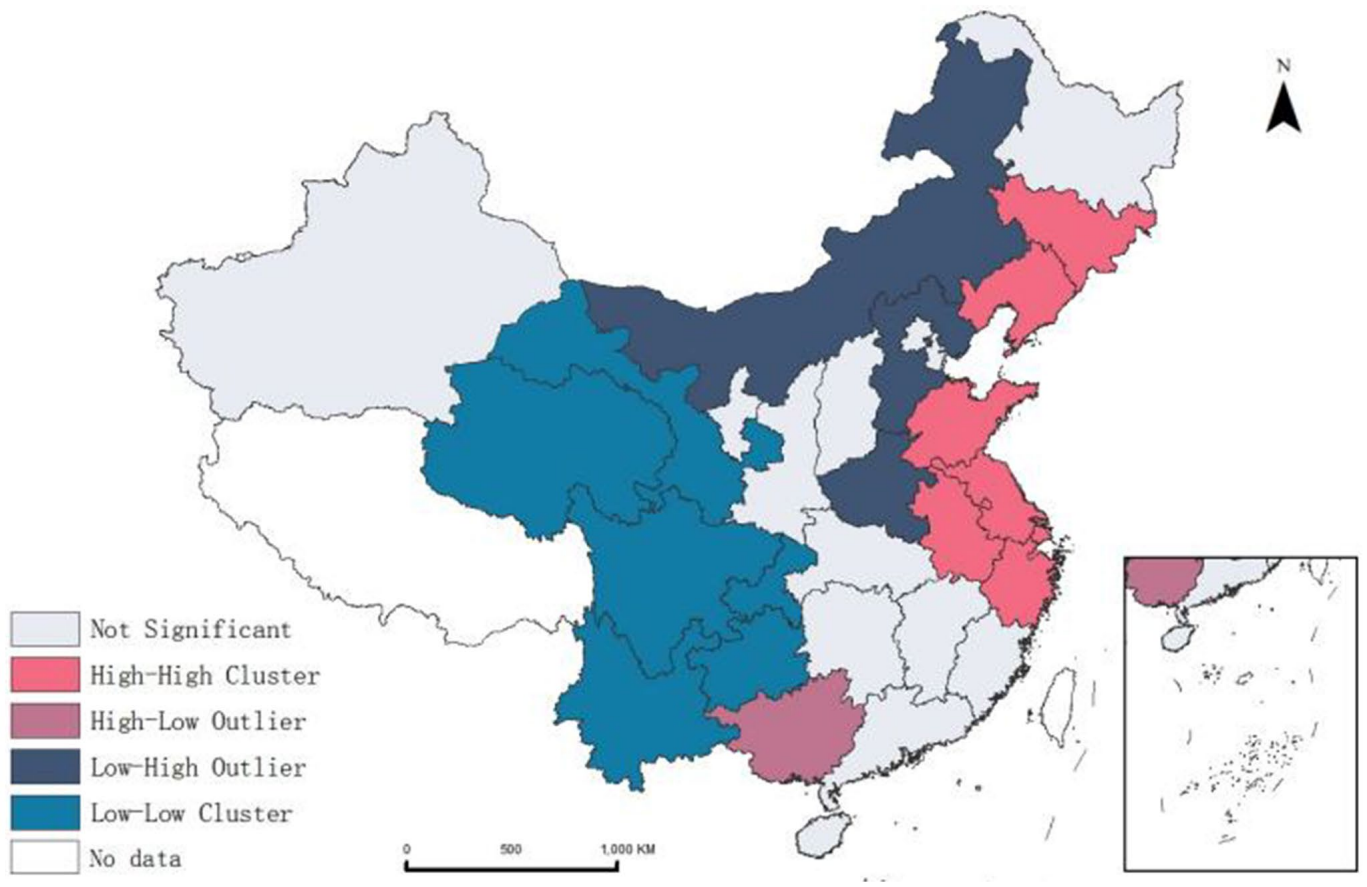
Spatial autocorrelation results and LISA clustering of the NT-ratio.

Using ArcGIS 10.8.1, we performed spatial autocorrelation analysis the NT-ratio across provinces, obtaining a positive Moran’s *I* of 0.278, revealing spatial dependence in migrants’ medical non-attendance post-illness. LISA clustering and mapping (Figure 5) further illustrated these patterns, identifying high-high clusters in the Northeastern (Jilin, Liaoning) and Eastern (Shandong, Jiangsu, Anhui, Zhejiang, Shanghai) regions, and low-low clusters in the Northwest (Gansu, Qinghai) and Southwest (Sichuan, Chongqing, Yunnan, Guizhou). This suggests that migrant patients’ utilization of health services exhibits spatial heterogeneity.

### 3.3 Hotspot analysis

**Figure 6:**
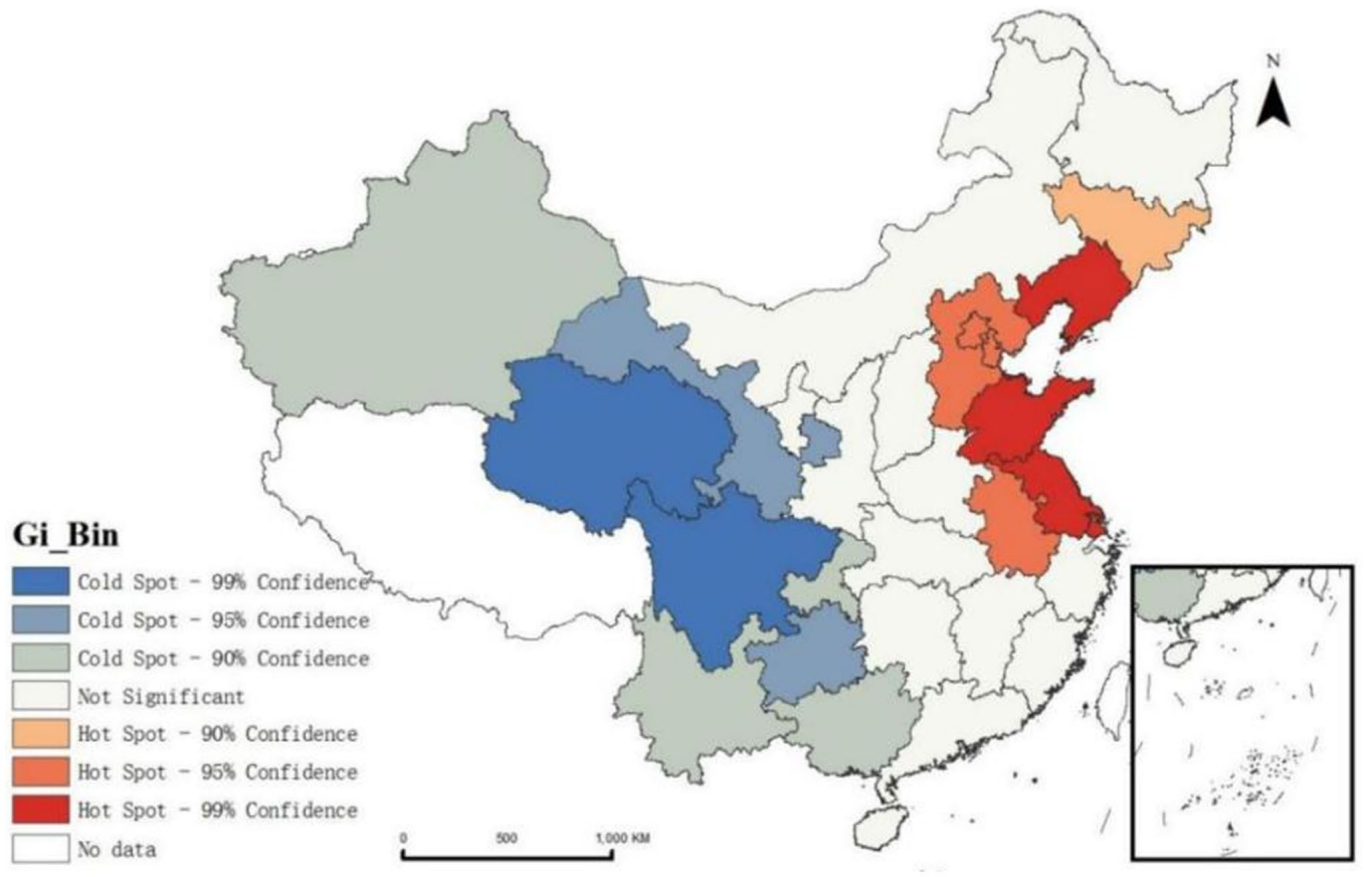
The pattern of cold spots and hot spots in the distribution of NT-ratio.

We applied the Getis-Ord Gi* method to analyze the NT-ratio across Chinese province. The results with 95% confidence (z-score greater than 1.96, probability value <0.05), show that the overall situation in the western region is better than that in the eastern region. Liaoning, Shandong and Jiangsu provinces were identified as significant hot spots, where the proportion of untreated migrants is highly clustered and shows signs of spreading to surrounding provinces (Figure 6).

Further examination of the migration patterns in these three provinces reveals that Liaoning and Jiangsu predominantly experience an influx of population from outside the province, accounting for 63.05 and 62.97% respectively, whereas Shandong sees mainly intra-provincial movement, with a smaller proportion of people coming from outside the province, at 23.86%. This suggests that the NT-ratio in Liaoning and Jiangsu is more closely linked with inter-provincial migration. These provinces should closely monitor health service utilization among incoming migrants, develop tailored policies, and strengthen management of health services to ensure migrants have access to appropriate medical care.

### 3.4 Geodetector analysis results

Spatial autocorrelation and hotspot analysis reveal a robust spatial correlation in the NT-ratio of the mobile population across provinces. To quantitatively analyze the spatial drivers and their intensity affecting NT-ratio distribution, we employed a geographical detector to examine risk factors at both the contextual characteristic (macro) and individual characteristic (micro).

#### 3.4.1 Contextual characteristics factors detection results

Number of Hospitals (NH) and Number of Hospital Beds (NHB) exhibit the highest explanatory power, exceeding 60%. Similarly, Number of Basic Medical Institutions (NBMI) and Number of General Practitioners (NGP) within the same category demonstrates substantial explanatory power, over 50% (Table 5), highlighting the role of medical resource distribution in migrants’ access to health service services. It is important to note that the issue of medical resources here does not merely refer to the insufficient total medical resources affecting the utilization of healthcare services by the migrants. Research has shown that individuals with lower age, income, and education levels typically have lower health awareness, making them more likely to choose healthcare utilization that are closer or less costly (42). Specific behavioral patterns of the China inner migrants, such as preferring to avoid complex medical procedures and holding stereotypes about the high costs and long wait times at large urban hospitals, can limit their opportunities to access high-quality medical resources. Even if the total number of hospital facilities is sufficient, the location and quality of services can still impact the health outcomes of migrant patients. Therefore, integrating medical resources requires not only an increase in quantity but also the equitable and rational distribution of high-quality medical facilities, particularly in suburban areas where low-income or migrants are concentrated.

**Table 5:** Significant factors of the individual characteristic dimension and explanatory power.

Rank	Variable	*q* statistic
1	Number of Hospitals (NH)	0.645***
2	Number of Hospital Beds (NHB)	0.645***
3	Number of basic medical institutions (NBMI)	0.600**
4	Number of general practitioners (NGP)	0.582***
5	participants in health education (PHE)	0.572**
6	Provincial annual precipitation (PAP)	0.456***
7	Population life expectancy (PLE)	0.446*
8	Provincial mortality rate (PMR)	0.380**
9	*Per capita* disposable income (PCDI)	0.331***
10	*Per capita* of Total Health Expenditure (PTHE)	0.229**
11	Urban employees insurance coverage rate (UEBMI)	0.201***
12	Industrial Waste Water Discharged (IWWD)	0.192***
13	Household Waste Water Discharged (HWWD)	0.095*

*** *p* < 0.001; ** *p* < 0.01; * *p* < 0.05 significance test.

**Figure 7:**
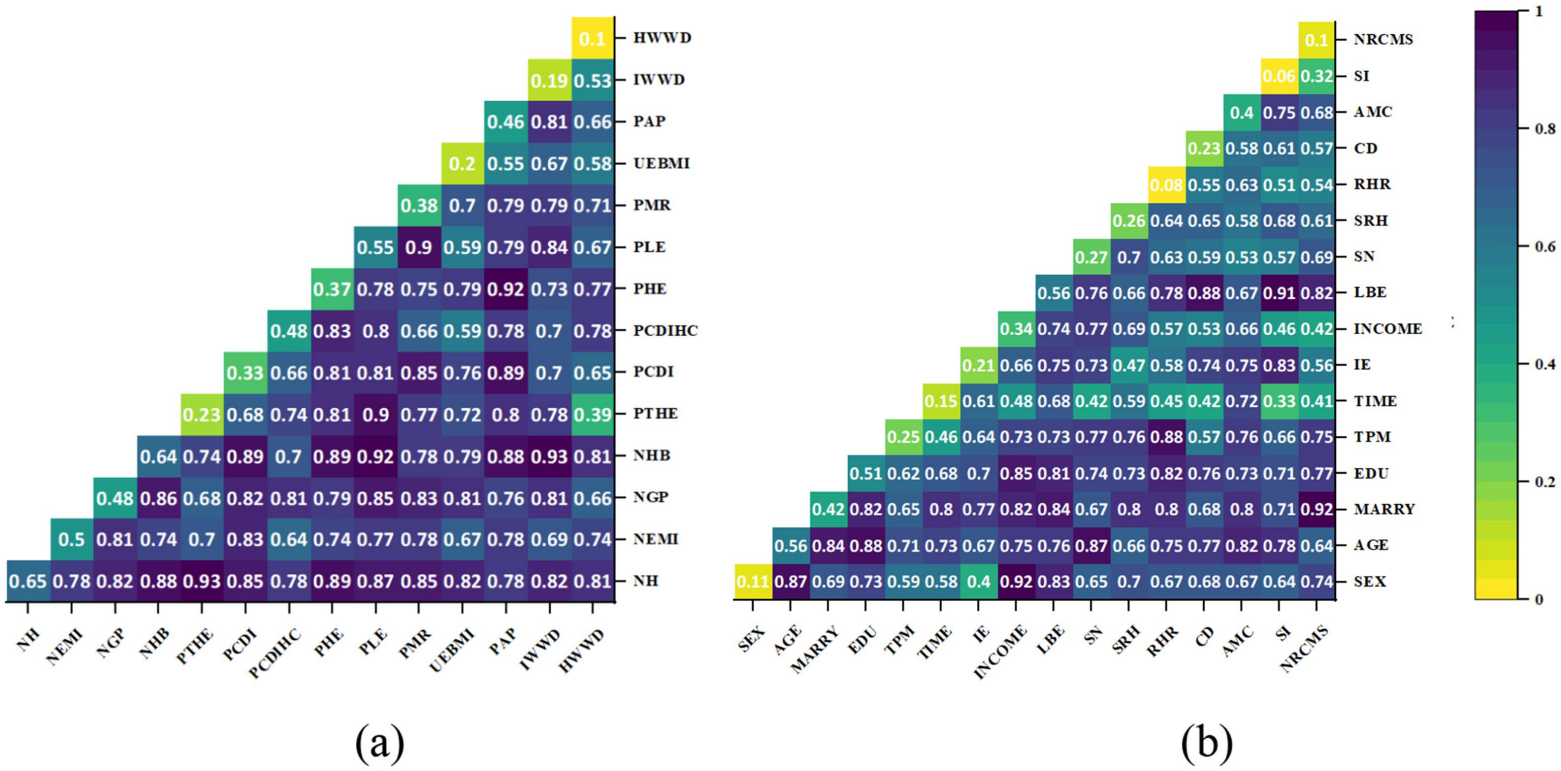
Interaction detector analysis results. **(a)** Contextual characteristics factors; **(b)** Individual characteristics factors.

Another factor with explanatory power exceeding 50% is Participants in Health Education (PHE), which, when interacted with NH and NHB, *q*(*X_PHE_*∩*X_NH_*) and *q*(*X_PHE_*∩*X_NHB_*) as high as 0.89 (Figure 7a). This suggests that actively engaging in health education through existing social networks enhances health literacy, enabling individuals to make healthier decisions within medical and social contexts. As previously mentioned for the migrants, better health literacy signifies possessing a valuable resource conducive to health (43). It can reduce the health detriments associated with mobility, enable rapid adaptation to the changes in the medical environment experienced during the migration process, and thus maintaining or enhancing health levels.

#### 3.4.2 Individual characteristics factors detection results

In the individual dimension, AGE has the strongest explanatory power (56.2%), interacting with Average years of education (EDU) and Social Network (SN), it shows a double enhancement effect (*q*(*X_AGE_*∩*X_EDU_*) and *q*(*X_AGE_*∩*X_SN_*) exceeding 85%) (Figure 7b). The Health Belief Model (HBM) proposes that individuals’ health behavior decisions are determined by their perceptions of threat severity, personal susceptibility to health issues, advantages of preventive actions, and obstacles faced. Specifically, older migrants with restricted educational backgrounds often undervalue both health risks and their own vulnerability (44, 45). Furthermore, the Social Determinants of Health (SDH) framework underscores how an individual’s environment impacts health outcomes. Therefore, limited education and advancing age may hinder their ability to effectively establish and utilize these networks.

An essential finding of this study is that the factor of LBE drives the tendency among the migrant population to forego treatment during adulthood. The explanatory power of LBE on the dependent variable NT-ratio exceeds 55%, validating the framework proposed in Figure 2. Although existing research has confirmed the Left Behind Experience (LBE)'s long-term negative impacts on physical and psychological well-being (46, 47)，little attention has been paid to the health service utilization of the migrant population with such experiences in adulthood. Our findings highlight that LBE significantly increases the likelihood of avoiding treatment post-illness. Although SI alone does not markedly affect the NT-ratio, its interaction with LBE reveals a nonlinear enhancement effect (*q*(*X_LBE_*∩*X_SI_*) reaching 91.2%), emphasizing Social integration (SI) as a pivotal mediator between LBE and health service avoidance, intensifying this avoidance tendency. Furthermore, the interaction of LBE with Chronic Disease (CD) awareness (*q*(*X_LBE_*∩*X_CD_*) at 88.2%) suggests that migrants, particularly those without or unaware of chronic conditions, may perceive their health as relatively stable, deterring them from seeking medical attention. The explanatory power of EDU on the NT-ratio also exceeds 50%, which aligns with the findings of studies on health service utilization across various countries worldwide (48, 49). Regarding interaction effects, besides AGE, *q*(*X_EDU_*∩*X_INCOME_*), also shows a high explanatory power (84.6%) (Figure 7a). Education level is a crucial factor affecting human capital, which is understandable. When migrant populations have lower levels of education and personal income due to economic constraints and limited health literacy, they are significantly more likely to choose the highly irrational health service utilization of not seeking treatment after falling ill (Table 6).

**Table 6:** Significant factors of the contextual characteristic dimension and explanatory power.

Rink	Variable	*q* statistic
1	Average age of migrant population (AGE)	0.562***
2	Left Behind Experience (LBE)	0.555***
3	Average years of education (EDU)	0.506***
4	Health Education (HE)	0.440**
5	Marital status (MARRY)	0.422***
6	Accessibility to Medical Care (AMC)	0.402**
7	Monthly income (INCOME)	0.345***
8	Social Network (SN)	0.266***
9	Self-rated Health (SRH)	0.263***
10	Trans-provincial migrant (TPM)	0.252***
11	Chronic Disease (CD)	0.233***
12	Income expenditure ratio (IE)	0.212*
13	Inflow time (TIME)	0.148**
14	Proportion of male migrant population (SEX)	0.112***
15	New rural cooperative medical service (NRCMS)	0.101**
16	Resident Health Record (RHR)	0.084***
17	Social integration (SI)	0.059***

*** *p* < 0.001; ** *p* < 0.01; * *p* < 0.05 significance test.

## 4 Discussion

The increasingly severe phenomenon of health inequities on a global scale indicates an urgent need for targeted research to halt this disturbing trend. This urgency compels us to shift our analytical lens from traditional epidemiological approaches to more nuanced, spatially-aware methods that offer deeper insights into the complexities of health service access and utilization. In contrast to previous focuses on individual differences among migrant populations, we first derives a model of health service utilization for migrant populations, analyze the health service services utilization of migrant populations after falling ill, spatial differentiation, and driving factors from a geographical spatial dimension. And to our knowledge, this is the first time the factor of left-behind experience has been incorporated into a model of health service utilization, using spatial statistical techniques to explore the medical behavior of migrant populations, providing a comprehensive picture of this topic. This study can summarize three main findings.

Firstly, migrants who opt for institutional treatment show significantly higher participation in any form of health education than those choosing pharmacy treatment or no treatment, emphasizing the role of health literacy (50). Similar results are observed among international migrants in Western countries such as Portugal (51) and Sweden (52). Many migrants have insufficient overall health literacy, particularly in healthcare access, disease prevention, and health promotion. This highlights the importance of promoting equitable access to health information, which helps reduce disparities in health access among migrant populations. Local pharmacies are the primary choice for health service after falling ill, accounting for 31.3% of cases; Meanwhile, 17.70% of individuals chose not to seek treatment, with these two groups combined accounting for over half of the population. Research from Europe, the U.S., and other regions revealing similar barriers, such as limited access to healthcare due to system (53, 54), language barriers, and cultural differences (55, 56), which affect migrants’ ability to seek timely and appropriate care. Our study reveals that internal migrants encounter similar obstacles. A significant portion of migrants either avoid formal healthcare or opt for pharmacies and self-treatment due to socioeconomic constraints and perceptions of cost and complexity within medical institutions.

Secondly, spatial autocorrelation and hotspot analysis of the NT-ratio on a national scale reveal a positive spatial correlation in the distribution of the migrants not seeking treatment. We attribute these regional disparities to two primary reasons. First, longstanding migration patterns from economically underdeveloped areas to economically developed areas, particularly from the central and western China to the eastern coastal regions，have led to large and dense populations in the eastern regions, which can easily lead to insufficient medical resources. Second, healthcare system shows significant policy and regional variance particularly in medical insurance types and reimbursement rates. For migrants, seeking medical treatment in the destination area often involves required procedures which some migrants are not bothered to do. Coupled with their limited education and income, and the stereotype of medical institutions being expensive, they often choose not to seek treatment after falling ill, an irrational and high-risk health service behavior. This pattern of healthcare avoidance among domestic migrants in China echoes challenges faced by migrants worldwide. Globally, migration often involves moving from less developed areas to more urbanized regions. Lessons from countries with more established migrant healthcare frameworks, such as Canada and Sweden, highlight the value of simplifying healthcare access through policies that streamline insurance procedures and promote health literacy (52, 57). Improving these aspects in China and elsewhere could help reduce healthcare inequalities and ensure that migrant populations can seek timely and affordable care. These include not only the redistribution of medical resources but also the implementation of culturally appropriate health education programs. In areas with high concentrations of migrant populations, especially in the aforementioned high NT-ratio clusters and hotspot regions, community workers and health service providers should promote policies and procedures for off-site medical insurance settlement through the internet, brochures, apps, and other channels to promote equal health service utilization for the migrant population.

Third, the factor detection by the geodetector indicate that age, left-behind experience, and educational level are the main individual factors affecting the NT-ratio across provinces. Among these, age has the greatest explanatory power, and there is a significant bidirectional enhancement effect on the NT-ratio when interacting with educational level and social relationships. All regions of the world are nowadays experiencing population aging. Existing studies have shown that for the older adult migrant population, factors such as educational level can also influence their choice of medical institutions, with a tendency towards irrational selection and difficulties in obtaining reimbursement from off-site medical insurance (58–60). This aligns with the findings of our study. However, we further discovered that poor social relationships exacerbate this irrational choice, extending the applicability of our conclusions. Additionally, the proportion of the migrant population with experiences of being left behind in various provinces is a leading factor driving the NT-ratio, and its interaction with social integration factors significantly enhances the effect (*q* = 0.912), which also validates the derivation in Figure 1. In urban areas, the high cost of raising children contrasts sharply with rural settings. The migrant population typically contends with lower wages, extended working hours, and a lack of stable social welfare benefits. Consequently, many migrants opt to leave their children in the care of grandparents back in their rural hometowns. This arrangement results in a substantial number of left-behind children.” In recent years, the number of urban left behind children in China has continued to expand, increasing from 8.7 million in 2010 to 25.16 million by 2020 (61, 62). Internationally, the number of left-behind children is even more striking: 27% of children in the Philippines are categorized as left-behind (63), 37% in Ghana (64), 36% in Moldova, 39% in Georgia (65). This indicates that the split family model of “Parents Migrant – Children Left-behind” remains a common pattern in many countries. The health status of the large number of left-behind children, both during their childhood and adulthood, warrants significant attention. Although research on the impact of LBE on an individual’s health in adulthood is already abundant, such as LBE having cumulative adverse effects and time effects on self-rated health status (47), the risk of depression, and anxiety increases during adolescence (66). However, few studies have focused on the specific behaviors through which left-behind experiences affect health outcomes, especially health service-seeking behavior. This indicates that children currently experiencing being left behind require enhanced dissemination of and education in health knowledge. In paying attention to the health status of left-behind individuals, one should consider not only psychological and physiological factors but also factors related to social interaction.

Additionally, the health service utilization by internal migrants is similarly influenced by the distribution of medical resources. Since Julian Tudor Hart introduced the Inverse Care Law in 1971, the paradox in medical resource distribution has been widely recognized: globally, accessibility to medical care is often inversely related to the individual or community need, meaning those in greatest need of medical services are frequently those who receive the least. The Deprivation Amplification Theory further elucidates how initial inequalities exacerbate adverse conditions (67). Although there has been an overall increase in medical resources globally, the unequal distribution of high-quality medical resources and the insufficient capacity of primary health care facilities remain significant issues, especially in developing countries (68–70). Therefore, future efforts should focus on enhancing the equitable distribution of medical resources, particularly in communities with high concentrations of migrant populations. The promotion and refinement of a tiered diagnosis and treatment system, along with the integration of resources from health, education, and social security sectors, are crucial to improving the health service utilization rate among migrant populations and promoting health equity.

## 5 Conclusion

This study has thoroughly analyzed the health service utilization among internal migrants in China, revealing a significant positive correlation with geographical locations. Our findings illuminate stark disparities in access to healthcare services, underpinned by both environmental and individual factors including age, left-behind experiences, health education, and the availability of high-quality medical facilities. These disparities highlight the need for focused interventions to improve health equity within migrant populations.

The challenges faced by internal migrants in China mirror global issues in migrant health, suggesting that the barriers to healthcare access are a widespread concern affecting many nations. By employing advanced methodologies including spatial autocorrelation and geodetector techniques, this study not only addresses specific local issues but also contributes to a broader understanding applicable in diverse international contexts. These methods offer powerful tools for other researchers seeking to analyze similar issues in different geographic settings, providing a valuable framework for comparative health policy research.

To address these challenges, we recommend increasing health education efforts targeted at the migrant population, with a particular focus on the older adult and special groups who have experienced being left behind. Additionally, optimizing the allocation of resources in primary healthcare institutions, especially in communities with high concentrations of migrants, is essential. Implementing a tiered diagnosis and treatment system, where patients are directed to appropriate care levels based on the severity of their conditions, along with fostering collaboration across the health, education, and social security sectors, are crucial steps towards improving health service utilization among migrant populations. These measures aim not only to bridge the current gaps but also to promote a more inclusive and equitable health system.

In conclusion, our study extends beyond identifying the unique challenges faced by China’s internal migrants to suggest practical and actionable strategies that could be adapted globally. Aligning our findings with international public health objectives underscores the necessity for comprehensive, cross-sectoral approaches to healthcare that accommodate the unique needs of all migrant populations. These measures aim not only to improve health outcomes among migrants but also to ensure that health equity is achieved as part of broader global health advancements.

### 5.1 Limitation

Due to the high mobility and large base of China’s migrant population, obtaining continuous tracking data presents challenges. Thus, this study utilized cross-sectional data from the 2017 China Migrants Dynamic Survey (CMDS), Although this dataset includes a broader range of survey items than the most recent 2018 data, it is essential for future research to explore longitudinal data on the migrant population. Additionally, while this study examined the impact of geographic environmental factors on the health service utilization among migrants and the resulting inequalities, it did not accurately capture the geographic locations and grid data of medical resources across provinces. Future research should aim to identify and assess the equity of medical resource distribution and uncover blind spots in service coverage across different regions, to more effectively adjust and optimize resource allocation.

## Data Availability

The datasets presented in this study can be found in online repositories. The names of the repository/repositories and accession number(s) can be found at: https://www.geodata.cn/wjw/#/home.

## Footnotes

^1^The policy primarily encompasses six aspects: establishing and improving health records for the migrant population, conducting health education for migrants, enhancing vaccination programs for migrant children, implementing infectious disease prevention and control measures for migrants, strengthening healthcare management for migrant pregnant women and children, and providing basic public family planning services for the migrant population.

^2^In China, Left-behind children refer to minors under the age of sixteen whose parents both work away from home or one works away while the other lacks guardianship capabilities. The experience of being left-behind refers to having the experience of being a left-behind child.

^3^The Relationship Between Mental Health and Child Abuse and Their Difference Between Left-at-Home Children and Those Living with Their Parents in Rural Areas: An Analysis Based on the Two-Dimensional Four-Fact Mental Health Structure.

^4^The Heihe-Tengchong Line, also known as the Hu Line, is an imaginary line that divides China into two regions with starkly contrasting population densities. It stretches diagonally from Heihe in the northeast to Tengchong in the south. As of 2015, the area east of the line, which makes up 43% of China’s land, is home to 94% of its population. Conversely, the western side accounts for 57% of the territory but only houses 6% of the population. This line has significant implications for China’s demographics, economy, and resource distribution.
